# Reduced Plasma Magnesium Levels in Type-1 Diabetes Associate with Prothrombotic Changes in Fibrin Clotting and Fibrinolysis

**DOI:** 10.1055/s-0039-3402808

**Published:** 2020-01-03

**Authors:** Amélie I. S. Sobczak, Fladia A. Phoenix, Samantha J. Pitt, Ramzi A. Ajjan, Alan J. Stewart

**Affiliations:** 1 School of Medicine, University of St Andrews, St Andrews, United Kingdom; 2 Leeds Institute of Cardiovascular and Metabolic Medicine, University of Leeds, Leeds, United Kingdom

**Keywords:** clot lysis, coagulation, metal homeostasis, plasminogen activator inhibitor-1, thrombosis

## Abstract

Individuals with type-1 diabetes mellitus (T1DM) have a higher risk of thrombosis and low plasma magnesium concentrations. As magnesium is a known regulator of fibrin network formation, we investigated potential associations between fibrin clot properties and plasma magnesium concentrations in 45 individuals with T1DM and 47 age- and sex-matched controls without diabetes. Fibrin clot characteristics were assessed using a validated turbidimetric assay and associations with plasma magnesium concentration were examined. Plasma concentrations of fibrinogen, plasminogen activator inhibitor-1 (PAI-1), and lipids were measured and fibrin fiber diameters assessed using scanning electron microscopy. Fibrin clot maximum absorbance was unchanged in subjects with T1DM compared with controls, while lysis time was prolonged ( *p* = 0.0273). No differences in fibrin fiber diameters or in lipid profile were observed between T1DM and controls. PAI-1 concentration was lower in the T1DM group compared with the controls ( *p* = 0.0232) and positively correlated with lysis time ( *p* = 0.0023). Plasma magnesium concentration was lower in the T1DM group compared with controls ( *p* < 0.0001). Magnesium concentration negatively correlated with clot maximum absorbance ( *p* = 0.0215) and lysis time ( *p* = 0.0464). A turbidimetric fibrin clot lysis assay performed in a purified system that included PAI-1 and 0 to 3.2 mM Mg ^2+^ showed a shortening of lysis time with increasing Mg ^2+^ concentrations ( *p* = 0.0004). Our findings reveal that plasma magnesium concentration is associated with changes in fibrin clot and lysis parameters.

## Introduction

Type-1 diabetes mellitus (T1DM) is a disease state in which the immune system of an individual destroys the β cells of the pancreas consequently affecting insulin secretion, resulting in raised glucose levels. 1 The incidence of T1DM varies widely across the world, with age-adjusted incidences ranging from 0.1/100,000 individuals per year in China and Venezuela to 36.5 and 36.8/100,000 individuals per year in Finland and Sardinia, respectively. 2 If T1DM is not correctly managed, the reduced insulin concentration in the blood can cause death, while the resultant elevated glucose concentration will increase the risk of microvascular complications and the risk of developing long-term vascular diseases. 3 Individuals with T1DM are estimated to have a standardized mortality ratio attributable to cardiovascular diseases of 5.7 for men and 11.3 for women compared with healthy individuals. 3 4 Despite the known increased risk of cardiovascular disease in individuals with T1DM, the pathophysiology underlying this relationship is not well understood.

Magnesium is an essential macronutrient and in its ionic form (Mg ^2+^ ) is essential for hemostasis and coagulation. 1 5 Mg ^2+^ is present in the blood at a concentration of 0.8 to 1.2 mM, 1 5 and is a required cofactor in approximately 600 enzymes and an activator for a further approximately 200 enzymes. 6 Mg ^2+^ is known to strongly influence the coagulation cascade. Indeed, addition of an excess of Mg ^2+^ results in an inability of blood to clot, 7 and although it is not used routinely, magnesium sulfate has been successfully used during blood collection as an alternative to citrate and ethylenediaminetetraacetic acid. 8 Mg ^2+^ homeostasis and the insulin concentration in the blood are closely linked. Notably, insulin affects tubular reabsorption of Mg ^2+^ from the blood by the kidney, while Mg ^2+^ is involved in energy metabolism and the regulation of insulin release. 9 In addition, magnesium deficiency in humans and animals can cause hypercoagulablity, 10 while magnesium deficiency in swine has been shown to lead to reduced plasma concentrations of coagulatory molecules including antithrombin, thromboxane, protein C, and endothelin-1. 11

In a recent study examining plasma concentrations of different metals in subjects with diabetes, we found that individuals with T1DM have a reduced plasma magnesium concentration compared with controls. 9 Given that patients with T1DM have an increased thrombotic risk, 12 and Mg ^2+^ is known to play an essential role in coagulation control, we hypothesized that the lowered plasma magnesium concentration associated with T1DM may affect blood coagulability. Here, we examine both fibrin clotting and clot lysis in plasma taken from the same T1DM and control cohort as previously reported, 9 and based on the results explore the relationship between plasma Mg ^2+^ concentration and measured fibrin clot and lysis parameters.

## Methods

### Clinical Sample Collection

Individuals with T1DM and controls were recruited from Leeds Teaching Hospital Trust following approval by the Leeds West Research Ethics Committee (REC: 09/H1307/12). Written informed consent to participate was obtained. A total of 45 individuals with T1DM and 47 controls were recruited from Leeds Teaching Hospital Trust. A total number of 92 samples is enough to detect 29.5% differences in individual fibrin clot parameters between groups, with a power of 80% (at *p* < 0.05), given the standard deviation of the studied variable at 50%. Exclusion criteria included: a history of acute coronary syndrome or stroke within 3 months of enrolment, prior treatment with aspirin, clopidogrel, warfarin, or nonsteroidal antiinflammatory drugs, current treatment with any drug other than insulin, a history of deep venous thrombosis or pulmonary embolism and previous or current history of upper gastrointestinal pathology, malignancy or coagulation disorders. Any individual with abnormal liver function tests (alanine transaminase >3 fold upper limit of normal) or abnormal thyroid function tests were not included. Finally, patients with proteinuria, advanced nephropathy, clinical signs of neuropathy, or retinopathy (except for those with background changes) were excluded. Written informed consent was obtained and baseline fasting blood samples were collected in trisodium citrate or lithium heparin coated tubes. Plasma was separated by centrifugation at 2,400 × g for 20 minutes at 4°C within 2 hours of collection. The samples were snap frozen in liquid nitrogen and stored at −40°C until analysis.

### Plasma Measurement of PAI-1, HbA1c, Lipid, and Lipoprotein Concentrations

The citrated plasma samples were used to measure the plasma fibrinogen concentrations using Clauss's method. The lithium-heparin plasma samples were used to measure the human serum albumin (HSA) levels with the bromocresol purple method using an automated analyzer (Architect; Abbot Diagnosis, Maidenhead, UK) and to measure the plasminogen activator inhibitor-1 (PAI-1) concentration with an enzyme-linked immunosorbent assay and the plasma concentrations of glycated hemoglobin A1c (HbA1c), triglycerides, cholesterol, high density lipoprotein (HDL), and low density lipoprotein (LDL) using routine methods. Total plasma zinc, copper, magnesium, and selenium concentrations were previously measured using the lithium-heparin samples by inductively coupled plasma-mass spectrometry on plasma samples (as described by Sobczak et al 9 ).

### Turbidimetric Fibrin Clot Formation and Lysis Assays in Plasma from Subjects with T1DM and Controls

To compare fibrin clot parameters in individuals with T1DM and controls, turbidimetric assays were performed as previously described. 13 14 Plasma samples from individuals with T1DM and from controls were thawed in a water bath at 37°C and centrifuged for 30 seconds at 3,600 × g. The samples were placed into a 96-well plate and then tPA (tissue plasminogen activator; Technoclone, Vienna, Austria) was added followed by thrombin (Calbiochem; San Diego, CA, USA) and CaCl _2_ . The final concentrations were: plasma diluted sixfold in buffer (50 mM Tris, 100 mM NaCl, pH 7.4), 3.75 mM CaCl _2_ , 0.03 U/mL thrombin, and 20.8 ng/mL tPA. The absorbance at 340 nm was read with a Multiskan FC plate reader (Thermo Scientific; Paisley, UK) every 12 seconds at 37°C. Clot formation and lysis parameters (maximum absorbance and lysis time defined as the time between maximum clotting and 50% lysis) were calculated from the resultant data with Prism 7.0 (GraphPad Software; La Jolla, CA, USA).

### Scanning Electron Microscopy

To assess fibrin fiber thickness in T1DM and controls, clots were formed in duplicate in the pierced lids of 0.6 mL centrifuge tubes in 45 µL volumes of pooled plasma diluted onefold in buffer (50 mM Tris, 100 mM NaCl, pH 7.4). The plasma samples were pooled from six randomly chosen subjects from each of the T1DM and control group. Clotting was induced by addition of 5 µL of 25 mM CaCl _2_ and 5 U/mL thrombin in buffer (50 mM Tris, 100 mM NaCl, pH 7.4). The clots were incubated for 2 hours at 100% humidity. For fixation, clots were given three washes (for 40 minutes each) with 67 mM sodium cacodylate, pH 7.4 and an overnight wash in 2% glutaraldehyde in sodium cacodylate buffer. Samples were dehydrated using a series of acetone washes and dried in a critical point drier. The clots were mounted on SEM stubs, coated with a 4 nm layer of iridium and viewed and photographed at ×10,000 magnification using a SU8230 scanning electron microscope (Hitachi; Maidenhead, UK) operating at 10 kV. Five images per sample were acquired and they were analyzed with Adobe Photoshop (Adobe Systems; San Jose, CA, United States); the diameters of 50 fibers per picture were measured. The images were adjusted for brightness and contrast and cropped.

### Turbidimetric Fibrin Clot Lysis Assays in a Purified System

To test the influence of the interaction between Mg ^2+^ and PAI-1 on lysis time, turbidimetric fibrin clot lysis assays were performed in a purified system that included exogenous PAI-1. A buffer (50 mM Tris, 100 mM NaCl, pH 7.4) was added to the wells of a 96-well plate, followed by fibrinogen (depleted in plasminogen, von Willebrand factor and fibronectin; Enzyme Research Laboratories, Swansea, UK). MgCl _2_ was then added followed by PAI-1 (Sigma-Aldrich; Gillingham, UK), then plasminogen (Stratech; Ely, UK) and tPA, and finally CaCl _2_ and thrombin. Final concentrations were 0.5 mg/mL fibrinogen, 2.5 mM CaCl _2_ , 0.05 U/mL thrombin, 3.12 µg/mL plasminogen, 39 ng/mL t-PA, 0 or 200 ng/mL PAI-1, and 0 to 3.2 mM MgCl _2_ . Absorbance at 340 nm was read with a Multiskan FC plate reader every 12 seconds at 37°C. Clot lysis was calculated from the resultant data using Prism 7.0 (GraphPad Software).

### Data Analysis and Representation

Distribution of the data was confirmed with normality tests and statistical differences between groups analyzed using either one-way analysis of variance or multiple Student's *t* -tests or, for continuous parameters, using Pearson's correlation test. Significance threshold was set at *p* ≤ 0.05. Statistical analyses were performed and graphs were generated with Prism 7.0 (GraphPad Software). Data are represented as mean ± standard deviation.

## Results

### Demographic Information and Measure of Plasma Concentrations of Different Molecules

Alterations in fibrin clot formation and lysis parameters were assessed between individuals with T1DM and controls. Demographic information was collected and the plasma concentrations of triglycerides, cholesterol, LDL, HDL, cholesterol ratio, HbA1c, HSA, and fibrinogen were measured as presented in Table 1 . The two study groups were matched for age and sex. The T1DM group had a higher BMI ( *p* = 0.0206), but no differences were observed in lipid concentrations (triglycerides, cholesterol, LDL, HDL, and cholesterol ratio). HbA1c was higher in the T1DM group ( *p* < 0.0001) compared with controls, while HSA concentrations were lower in the T1DM group ( *p* = 0.0269) compared with controls and fibrinogen concentrations were comparable between groups.

**Table 1:** Demographic information and measures of plasma concentrations of relevant molecules for the clinical cohort

	Controls ( *n* = 47)		Subjects with T1DM ( *n* = 45)		Statistical significance
	Mean	Standard deviation	Mean	Standard deviation	*p* -values
Age (years)	24.3	6.2	26.3	6.8	0.1436
Sex (% of male)	55	–	58	–	0.8145
Height (m)	1.73	0.09	1.73	0.10	0.7594
BMI (kg/m ^2^ )	23.0	2.9	24.6	3.6	0.0206
Numbers of smoking individuals	2	–	13	–	–
Numbers of macrovascular events	0	–	0	–	–
Numbers of microvascular events	0	–	1	–	–
Numbers of individuals with familial history of autoimmunity	12	–	16	–	–
Numbers of individuals with familial history of Huntington's disease	16	–	9	–	–
Diabetes duration (mo)	–	–	124.8	101.8	–
HbA1c (mmol/mol)	34.6	3.1	72.6	17.6	<0.0001
HbA1c (%)	5.3	0.3	8.8	1.6	–
Triglyceride (mmol/L)	0.9574	0.307	0.8953	0.3518	0.3738
Cholesterol (mmol/L)	4.23	0.9065	4.445	0.6899	0.2072
LDL (mmol/L)	2.364	0.7551	2.488	0.5446	0.3858
HDL (mmol/L)	1.455	0.3764	1.551	0.4137	0.2529
Cholesterol/HDL ratio	3.019	0.6943	2.964	0.6617	0.7045
HSA concentration (g/L)	45.9	2.4	44.7	2.6	0.0269
Fibrinogen (mg/mL)	2.28	0.47	2.39	0.46	0.2425

Abbreviations: BMI, body mass index; HbA1c, hemoglobin A1c; HDL, high density lipoprotein; HSA, human serum albumin; LDL, low density lipoprotein; T1DM, type-1 diabetes mellitus.

### Fibrin Clot Formation and Lysis Parameters and Fibrin Clot Ultrastructure

**Fig. 1:**
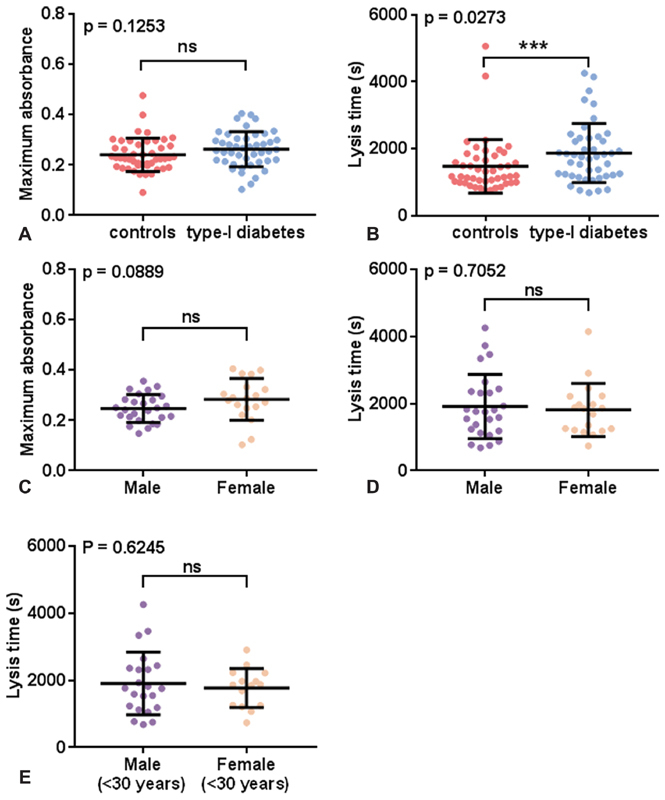
Comparison of fibrin clot parameters in plasma samples from individuals with T1DM and controls. ( **A, B** ) Fibrin clot parameters were compared between controls and T1DM, ( **C, D** ) between all males and females with T1DM and ( **E** ) between males and females under 30 years old with T1DM. Clot formation experiments were performed in plasma ( *n* = 47 for subjects with T1DM and *n* = 45 for controls). Plasma was diluted six-fold in buffer and the final concentrations of reagents used were: 7.5 mM CaCl _2_ , 0.03 U/mL thrombin, and 20.8 ng/mL tPA. ( **A, C** ) Maximum absorbance and (B, D, E) and lysis time were measured. Maximum absorbance was not different between the control and the T1DM groups. Lysis time was increased in individuals with T1DM compared with controls ( *p* = 0.0273). Maximum absorbance and lysis time were not different when comparing all males and females with T1DM or only those under 30 years old. Statistical significance is indicated with ns where *p* > 0.05 and *** where *p* < 0.001.

**Fig. 2:**
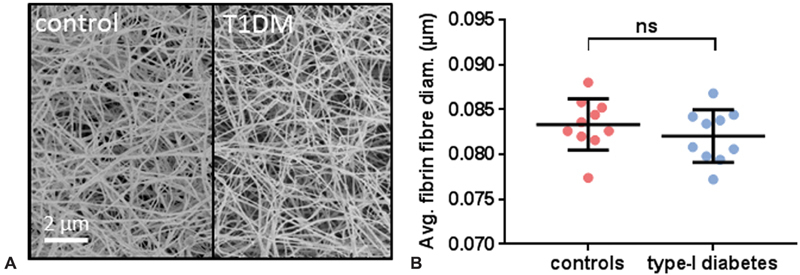
Comparison of fibrin fiber diameters in plasma from individuals with T1DM and controls. The clots were formed in duplicate with 45 µL of plasma (pooled from six random samples) diluted two-fold in buffer and 5 µL of 25 mM CaCl _2_ and 5 U/mL thrombin; five images were taken per clot and 50 fibers were measured per image. ( **A** ) Representative SEM images of a clot formed in plasma from the control group or from the T1DM group. ( **B** ) Average diameters of fibrin fibers in T1DM and control groups. No difference in the thickness of fibers was observed between the controls and the subjects with T1DM. ns indicates that *p* > 0.05. The images were adjusted for brightness and contrast and cropped.

Validated turbidimetric fibrin clot formation and lysis assays 13 14 were performed on the plasma samples taken from the T1DM and the control groups. The maximum absorbance of the fibrin clots did not differ between the T1DM and control groups ( Fig. 1A ). Lysis time was longer in the T1DM group compared with the control group ( *p* = 0.0273, Fig. 1B ). No differences between males and females were found in the T1DM group for maximum absorbance or lysis time, either in individuals of all ages ( Fig. 1C,D ) or in lysis time in individuals under 30 years old ( Fig. 1E ). SEM experiments were performed on pooled plasma (from both sexes) from the T1DM and the control groups and the average diameter of fibrin fibers were measured ( Fig. 2 ). The fiber diameter was not statistically significantly altered in the T1DM group compared with the control group.

### PAI-1 Concentration in Subjects with T1DM and Correlation with Lysis Time and Plasma Magnesium Concentration

**Fig. 3:**
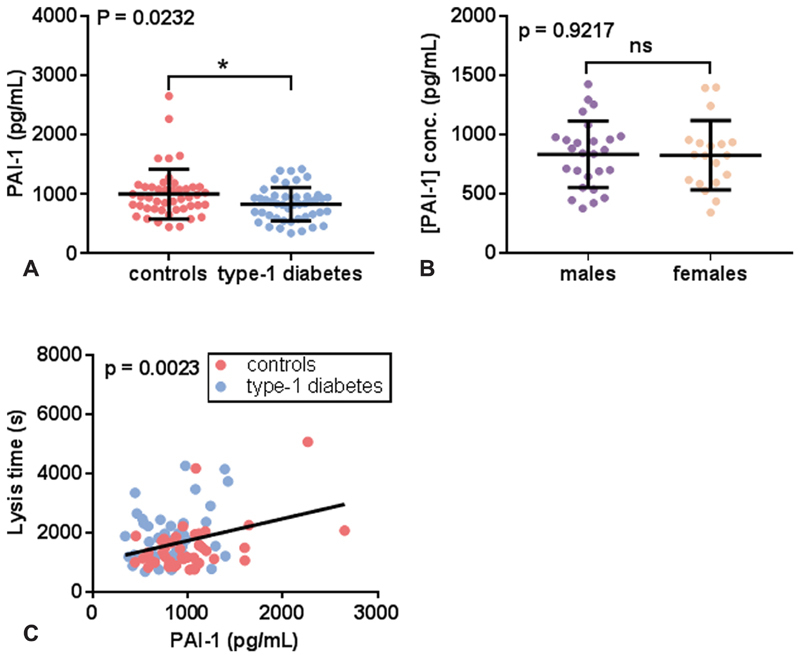
Relationship between lysis time and the plasma concentrations of PAI-1 in T1DM and controls. ( **A** ) Comparison of plasma PAI-1 concentrations between controls and subjects with T1DM. ( **B** ) Comparison of PAI-1 concentration between males and females with T1DM. ( **C** ) Relationship between lysis time and plasma PAI-1 concentration. PAI-1 concentration was lower in individuals with T1DM ( *p* = 0.032), but there was no difference between males and females with T1DM. Lysis time was positively correlated with PAI-1 concentration ( *p* = 0.0023). ns indicates that *p* > 0.05 and * where *p* < 0.05.

The plasma PAI-1 concentration was measured in the T1DM and control groups and potential associations with lysis time and plasma magnesium concentrations were examined ( Fig. 3 ). The PAI-1 concentration was lower in the T1DM group compared with the control group ( *p* = 0.0232), and PAI-1 concentration was found to be positively associated with lysis time ( *p* = 0.0023).

### Relationship between Total Plasma Concentrations of Magnesium and Clot Formation and Lysis Parameters in Subjects with T1DM

**Fig. 4:**
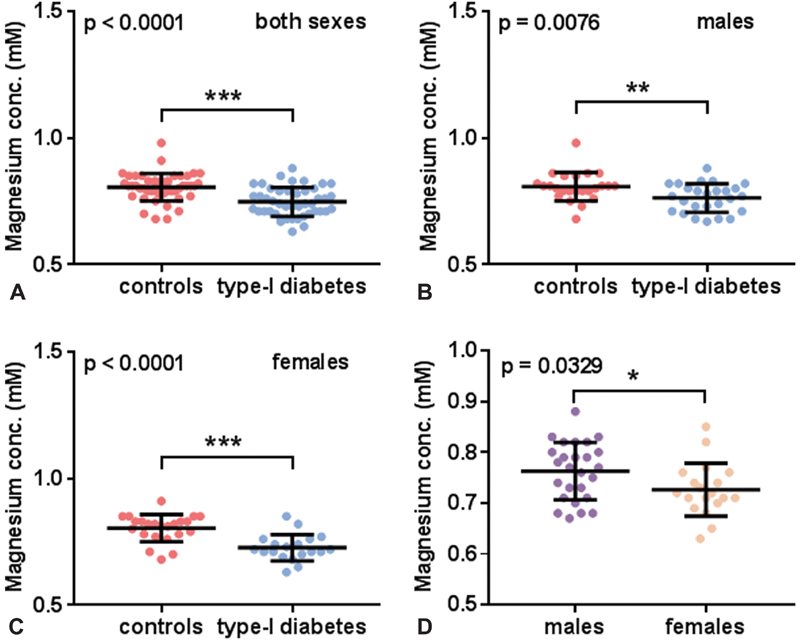
Comparison of total plasma magnesium concentration between individuals with T1DM and controls. Comparison of total plasma magnesium concentration between individual with T1DM and controls in ( **A** ) both sexes, ( **B** ) males, and ( **C** ) females. ( **D** ) Comparison of total plasma magnesium concentration between males and females with T1DM. Total plasma magnesium concentrations were lower in subjects with T1DM compared with controls when examining both sexes together and in both males and females when examined separately ( *p* < 0.0001, *p* = 0.0076, and *p* < 0.0001, respectively). Total plasma magnesium concentration was lower in females with T1DM compared with males with T1DM ( *p* = 0.0329). Statistical significance is indicated with * where *p* <0.05, ** where *p* <0.01, and *** where *p* <0.001. A summary of this data was published before but no detailed analysis was previously carried out. 9

**Fig. 5:**
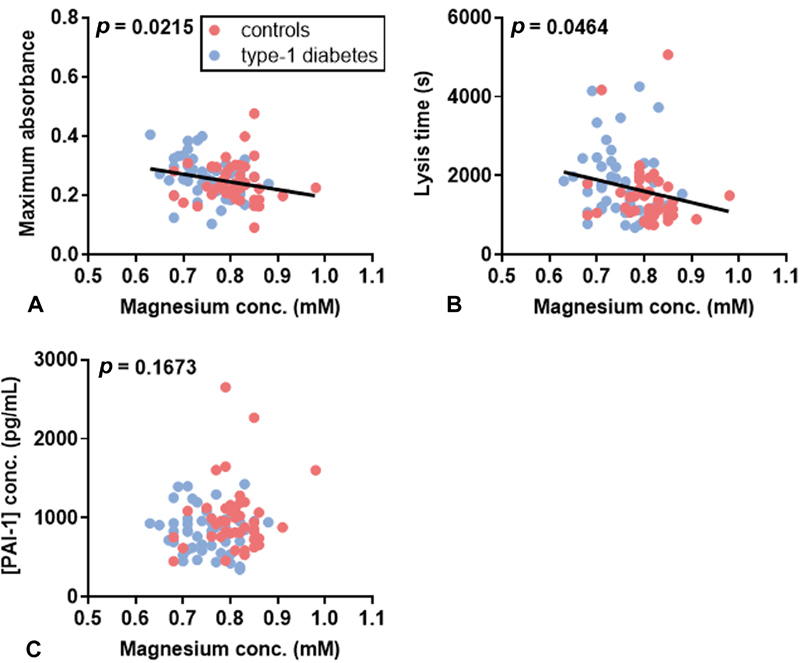
Relationship between total plasma magnesium concentration and fibrin clot parameters in T1DM subjects and controls. ( **A** ) Relationship between maximum absorbance and total plasma magnesium concentration. ( **B** ) Relationship between lysis time and total plasma magnesium concentration. ( **C** ) Relationship between PAI-1 concentration and total plasma magnesium concentration. Total plasma magnesium concentration negatively correlated with maximumabsorbance ( *p* =0.0215) and lysis time ( *p* = 0.0464), but did not correlate with PAI-1 concentration.

In a previous study, total plasma magnesium was measured in plasma taken from subjects with T1DM and age-matched controls using inductively coupled plasma-mass spectrometry ( Fig. 4) . 9 The mean total plasma magnesium concentration was lower in the T1DM group compared with the control group when assessing both sexes together ( *p* < 0.0001) or when looking separately at male subjects ( *p* = 0.0076) or female subjects ( *p* < 0.0001). The total plasma magnesium concentration was lower in females with T1DM than males with T1DM ( *p* = 0.0329). Potential associations between total plasma magnesium concentration and fibrin clot parameters or PAI-1 were then assessed ( Fig. 5 ). Negative correlations were found between total plasma magnesium concentration and maximum absorbance ( *p* = 0.0215) and between total plasma magnesium concentration and lysis ( *p* = 0.0464). However, the PAI-1 concentration did not correlate with the plasma magnesium concentration.

### Influence of Mg ^2+^ -PAI-1 Interaction on Lysis Time

**Fig. 6:**
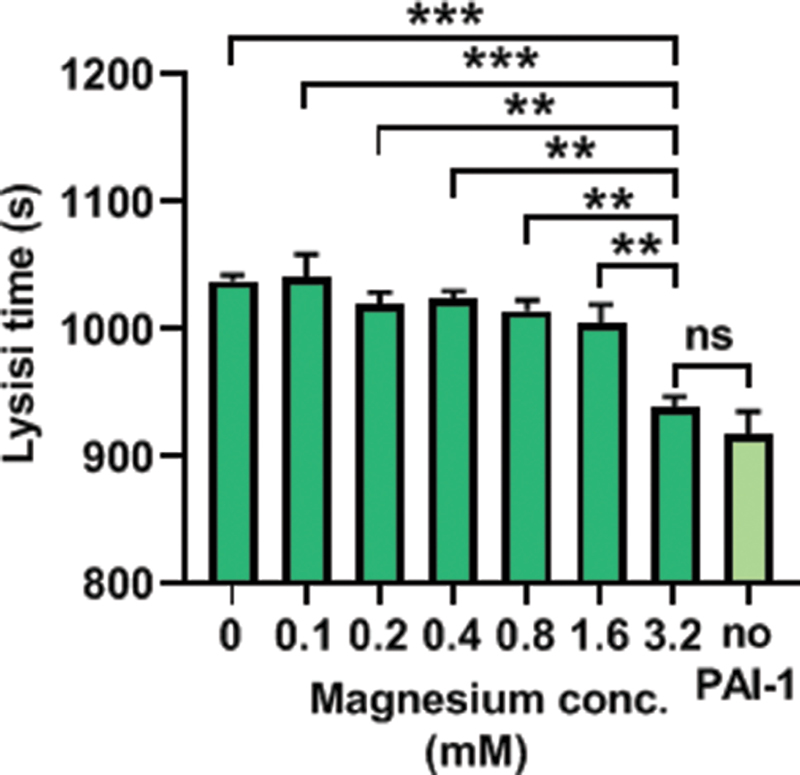
Effects of Mg ^2+^ - PAI-1 interactions on fibrin clot lysis. Turbidimetric assays were performed in a purified system made of buffer (50 mM Tris, 100 mM NaCl, pH 7.4), 0.5 mg/mL fibrinogen, 2.5 mM CaCl _2_ , 0.05 U/mL thrombin, 3.12 µg/mL plasminogen, 39 ng/mL t-PA, 0 or 200 ng/mL PAI-1, and 0 to 3.2 mM MgCl _2_ ; the lysis time was calculated. Addition of 0.1 to 3.2 mM Mg ^2+^ shortened lysis time ( *p* = 0.0004). In the presence of 3.2 mM Mg ^2+^ and PAI-1, lysis time was not significantly different to its value in the absence of PAI-1. ns indicates that *p* > 0.05, ** where *p* < 0.01, and *** where *p* < 0.001.

To assess the influence of the Mg ^2+^ -PAI-1 interaction on lysis time, turbidimetric fibrin clot lysis assays were performed in a purified system that included PAI-1 in the presence of 0 to 3.2 mM Mg ^2+^ ( Fig. 6 ). Lysis time was found to decrease with increasing Mg ^2+^ concentrations ( *p* = 0.0004). The lysis time obtained in the presence of each Mg ^2+^ concentration did not significantly differ except between 3.2 mM Mg ^2+^ and the other Mg ^2+^ concentrations, where it was found to be lower. In the presence of PAI-1 and 3.2 mM Mg ^2+^ , the lysis time was not significantly altered compared with the absence of PAI-1.

## Discussion

Individuals with T1DM are known to be deficient in magnesium and to have an elevated thrombosis risk. Therefore, we wanted to assess whether the plasma Mg ^2+^ concentration relates to fibrin clot formation and lysis as induced by the addition of thrombin, Ca ^2+^ , and tPA. To this end, clinical samples from individuals with T1DM and controls were examined. Both groups were matched in age and sex. The plasma HSA concentration was reduced in subjects with T1DM compared with the controls, which may be a result of insulin deficiency. 15 Insulin deficiency has also been associated with increased fibrinogen synthesis, probably as an acute-phase response, and indeed a slight but not significant increase in plasma fibrinogen concentration was observed. 15 In a study by Agren et al, fibrinogen concentration has previously been reported to be lower in individuals with T1DM compared with healthy controls, but this work was performed on an older cohort; the mean age of individuals with T1DM was 44 years in their study compared with 26 years in our study. 16

No difference was observed in fibrin fiber thickness between clots formed in pooled samples taken from individuals with T1DM and those taken from controls. The turbidimetric assay revealed that clots formed in plasma from individuals with T1DM exhibited an increase in lysis time, but an unchanged maximum absorbance compared with controls. A previous study by Tehrani et al reported that no differences in fibrin clot properties could be seen between males and females (aged 20 years or above) with T1DM, but those females with T1DM aged less than 30 years had less permeable fibrin clots and prolonged lysis times than age-matched males with T1DM. 17 This study did not compare clot properties between individuals with T1DM and healthy controls. 17 In the results presented here, no differences between males and females were observed, either when considering all the subjects with T1DM or only those aged less than 30 years old. The study by Agren et al also found no sex-specific differences in lysis time between individuals with T1DM and controls. 16 However, the study also reported shorter lysis times in individuals with T1DM, which they explained by the fibrin clots being more resistant to fibrinolysis (with more antiplasmin incorporated in the fibrin clots despite lower plasma concentrations), while the fibrinolytic potential of the plasma simultaneously increased (marked by a reduced activity of PAI-1) and the fibrinogen concentration was reduced. 16 The difference between these results and the lysis time observed in our study could be explained by the age of the patients (mean age 44 years in the Agren et al study and 24 years in this study) and the treatment regimen at the time of blood collection (in the Agren et al study, some patients were on ACE inhibitors, statins, and estrogens, while our study excluded them), resulting in unchanged fibrinogen concentrations between our T1DM cohort and control groups.

It was found that the plasma PAI-1 concentration was reduced in the T1DM group compared with the controls, which is consistent with previous observations showing reduced or similar PAI-1 levels in T1DM compared with controls. 14 16 However, this is contrary to a study by Adly et al, which reported elevated concentrations of PAI-1 in children and adolescents with T1DM—especially in those with microvascular complications—compared with controls. 18 This difference may be explained by the presence of complications in that study, whereas patients in our study had no clinically significant diabetes-associated complications. PAI-1 concentration positively correlated to lysis time in our study, which is in accord with its known importance in regulating the fibrinolytic process. 19 20

When examining potential associations between total plasma magnesium concentration and clot parameters, magnesium concentration was found to negatively correlate with maximum absorbance and with lysis time. Thus, the deficiency of magnesium found in the subjects with T1DM could potentially directly result in increased maximum absorbance and lysis time. Fibrinogen concentration was similar in T1DM and control groups and therefore cannot explain the significant difference observed in maximum absorbance. The reduced PAI-1 concentration in the T1DM group should result in increased tissue plasminogen activator activity and enhanced lysis, which is in opposition to our results. Thus, another factor must explain the increased in lysis time observed in the T1DM group. The negative correlation between total plasma magnesium concentration and lysis time may partly provide an explanation for this phenomenon.

Several studies have previously examined the effects of Mg ^2+^ on coagulation; however, some of the reported results are contradictory. This is likely because the effects of Mg ^2+^ on fibrin clot formation and lysis are complex, with Mg ^2+^ having the ability to influence many different interactions (both positively and negatively). In addition, in vitro fibrin clotting can be initiated at different points within the coagulation cascade. This is why in some studies initiation of clotting at certain points in the cascade may mean that parts of the cascade are bypassed; thus, the effect of Mg ^2+^ may differ depending on the experimental design. In our assays, fibrin clot formation was triggered by the addition of thrombin and Ca ^2+^ . This implicates both the intrinsic and extrinsic pathway of the coagulation cascade as thrombin does not only cleave fibrinogen into fibrin, but it also activates proteins participating in prothrombin activation, including factor VII, factor VIII, factor V, and factor XI. Thus, while the addition of thrombin to initiate coagulation signifies that the thrombin concentration will be the same when the clot starts to form, differences in plasma composition will affect how prothrombin is activated or inhibited and so thrombin concentration during later stages of clot formation and during clot lysis, which has been shown previously to affect fibrin clot parameters. 21 Nevertheless, our assay is limited by the absence of the endothelium, which prevents some interactions from taking place (in particular with the absence of the release of tissue factor and phospholipids). Another important factor in the fibrin clot experiments is the concentration of Mg ^2+^ used, as Mg ^2+^ potentiates interactions at lower concentration (<10 mM), while at high concentrations, (>10 mM, not physiological) it competes with Ca ^2+^ and interferes with its role as a cofactor. 7 22 In our assay an excess of Ca ^2+^ was added while no additional Mg ^2+^ was added, thus preventing Mg ^2+^ competition with Ca ^2+^ from becoming relevant. Due to the lack of specific indicators for Mg ^2+^ , it is impossible to know the amount of Mg ^2+^ ionized during recalcification of citrated plasma. Despite this, the dissociation constants of both Ca ^2+^ and Mg ^2+^ binding to citrate (in the same buffer; 5 mM Tris, 5mM MES, 5 mM PIPES, pH 6.0) have been measured. Under these conditions, it was shown that Ca ^2+^ binds citrate with a K _d_ of 66 µM while Mg ^2+^ binds citrate with a K _d_ of 157 µM, showing Mg ^2+^ to possess a lower affinity than Ca ^2+^ for citrate. 23 Thus, as a large excess of CaCl _2_ is added into the diluted citrated plasma, it can be expected that most Mg ^2+^ (>95%) will be ionized under the experimental conditions.

It has been shown previously that Mg ^2+^ (0.5–1 mM) accelerates the activation of factor X by activated factor IX (in the presence of activated factor VIII, phospholipids and Ca ^2+^ ) as well as the activation of factor IX and of factor X by the activated factor VIIa-tissue factor complex. 5 24 Furthermore, the addition of Mg ^2+^ in plasma from healthy subjects has been shown to have various effects on clotting time depending on the type of plasma and magnesium concentrations used in the studies and on the method by which clotting was induced. 22 24 25 Mg ^2+^ is also involved in anticoagulatory processes as 0.6 mM Mg ^2+^ potentiates the inactivation of activated factor V by activated protein C. 26 In addition, increasing Mg ^2+^ concentrations added to whole blood (0–8 mM Mg ^2+^ , no clotting occurred with 10 mM Mg ^2+^ ) have been shown to reduce the spontaneous fibrin lysis time, which was hypothesized to be due to its inhibition of PAI-1 in the presence of thrombin and vitronectin. 22 Thus, magnesium inhibition of PAI-1 could result in an increase in tPA activity, an increase in clot lysis and a shorter lysis time. This could explain why low plasma magnesium concentrations are correlated with increased lysis time. To assess the effect of Mg ^2+^ -PAI-1 interactions on lysis time, we performed a turbidimetric assay in a purified system incorporating PAI-1 and different Mg ^2+^ concentrations. The results confirmed a shortening of the lysis time by Mg ^2+^ . The presence of supraphysiological concentrations of Mg ^2+^ (ca. 3.2 mM) completely inhibited PAI-1 activity. However, the inhibitory effect was subtle at physiological Mg ^2+^ concentrations (ca. 0.6–1.0 mM) and so Mg ^2+^ -inhibition of PAI-1 is unlikely to fully justify the longer lysis time found in the T1DM group. Another unknown mechanism is most likely involved in further prolonging lysis time.

Alterations in clot lysis may also affect clot formation and explain the correlation between total plasma magnesium concentration and clot maximum absorbance; however, if this is the case, this did not translate to a difference in maximum absorbance in the T1DM group compared with the controls. Despite females with T1DM having higher risk of thrombosis than males with T1DM, no difference between the sexes could be seen in lysis time or clot time. 17 27 However, the total plasma magnesium concentration is lower in females with T1DM than in males with T1DM, so alterations in clot formation and lysis caused by magnesium may be more common in females; this correlates with the higher thrombotic risk observed in females (compared with males). It is of interest to note that magnesium supplementation in individuals with T1DM has been shown to decrease the risk of developing complications associated with diabetes, including cardiovascular disease. 27

## Conclusion

In this study, we demonstrate that plasma magnesium concentration negatively associates with both fibrin clot density (maximum absorbance) and with lysis time in subjects with T1DM and controls. Given that Mg ^2+^ is a known regulator of multiple proteins that facilitate clot formation and lysis, this observation may help to explain the higher risk of thrombosis observed in those with T1DM, as such individuals have lower plasma magnesium concentrations than those without diabetes. Collectively, the results presented here highlight the importance of Mg ^2+^ for normal clot formation and lysis and lends support to the hypothesis that lowered plasma Mg ^2+^ levels in T1DM contribute to the development of thrombotic complications.

## Notes

**What is known about this topic?**

- Individuals with T1DM are at a higher risk of developing microvascular complications and long-term vascular diseases.
- Plasma magnesium levels are lower in those with T1DM compared to individuals without diabetes.
- Magnesium is a known regulator of coagulation and fibrin network formation.

**What does this paper add?**

- A lowered plasma magnesium concentration is associated with increased fibrin clot density and delayed clot lysis.
- Mg ^2+^ -dependent inhibition of PAI-1 contributes to this phenomenon but is unlikely to be the only mechanism by which Mg ^2+^ influences fibrin clot formation and lysis.
